# Supplementary material for: A data-driven biology-based network model reproduces C. elegans premotor neural dynamics
Source: PLoS Comput Biol. 2025 Dec 29;21(12):e1013818. doi: 10.1371/journal.pcbi.1013818 (PMC12768384; doi:10.1371/journal.pcbi.1013818)
Supplement: Attachment — Submitted filename: Response to Reviewers.pdf [file pcbi.1013818.s001.pdf]

Dear Dr. Barbara Webb and Dr. Hugues Berry,
PLOS Computational Biology

RE: Manuscript # PCOMPBIOL-D-25-00009, “A data-driven biophysical network model reproduces *C. elegans* premotor neural dynamics”

We would like to take this opportunity to thank the reviewers for their time and effort in evaluating our manuscript. We also thank you for allowing us a chance to respond to the reviewers’ remarks and criticisms. Below you will find detailed comments regarding each remark made by the referees. Our answers to the remarks are in green, while text changes are in blue. We have included a copy of the revised manuscript with text changes in blue. This will allow the referees to quickly track the changes made to the manuscript upon their request. We have added four additional sections with four new figures showing new simulations and analysis. Overall, the manuscript quality has improved as a result of the comments and observations of the referees and we hope that it will be accepted for publication in PLOS Computational Biology.

Sincerely,

Megan Morrison and Lai-Sang Young

Reviewer 1

Major

1. The proposed model covers a major subset of the command interneurons, but other neuronal activities are treated as inputs to the model. If these inputs include clear signals that indicate forward or backward movement, it would be easy to switch between forward and backward movement even without the proposed model. In fact, the experimental data used by the authors (Atanas et al 2023) includes experiments in which aversive heat stimulation was applied to induce strong backward movement. It is necessary to examine the properties of the input signal in addition to the presence or absence of such thermal stimulation. For example, as in Figure 7 of Hallinen 2021 (doi: 10.7554/eLife.66135), it may be possible to examine how the neural activity in the model is related to the main components extracted by principal component analysis of the input signal. By separating whether the transitions between the forward and backward states in the model are strongly dependent on the input signal, it becomes easier to understand how the state transitions occur, and it seems to be a good example that shows the importance of the proposed model.

   Response:

We added a section analyzing how applying heat stimulus to the model, via heat responsive neurons, affects the probability of transitioning to a reversal state, as was done in Ananas & Kim et al. (2023).
Section added: "Replicating the impact of heat shocks". (pg. 14)
Figure added: Figure 8.

We also added section "Promoter and suppressor signal neurons" (pg. 11), in which we analyze how transitions between the forward and reversal states are dependent on input signals. (Figure 5)

In the spirit of the Reviewer's suggestion, we have also added sections "Ablation of core neurons" (pg. 11), and "Signal propagation map" (pg. 12).
Figures added: Figures 6, 7.

2. The authors claim that "The dynamics produced are complex: they are neither completely random as in the Markov chains models nor are they limit cycles as in earlier dynamical systems models." However, the dynamics of the proposed model are heavily dependent on external forces, so a superficial comparison is not valid. It is necessary to analyze and compare how state transitions occur in the model, independently of the model's input. I would like to see how the state transitions occur in the proposed model, after extracting the time periods when the effect of external forces is weak, as shown in comment 1). In addition, the differences in the roles of individual neurons are also interesting, but they have not been analyzed at all. For example, it is thought that RIM stabilizes the forward and backward states (Sordillo A., et al., 2021, eLife). Also, is AVD, which the authors incorporated into the model, important for switching between forward and backward? To investigate the role of these neurons, it may be useful to perform an ablation study of RIM and AVD in the model. I believe that such additional analysis is essential for the authors' main question, "How do these neurons work together to determine behavior, sustain different behaviors, and switch between them?"

Response:
In the added section "Signal propagation maps", we simulate the core neuron dynamics with perturbations applied between periods of no input. In the absence of input the core group converges to stead states (pg. 13).
In the absence of any stimulus the neurons stabilize at different values with the forward neurons more active than the reversal neurons. Perturbing each neuron induces changes to the state of downstream neurons, as shown in Figure 7(d), emulating the experimental results reproduced in Figure 7(c).

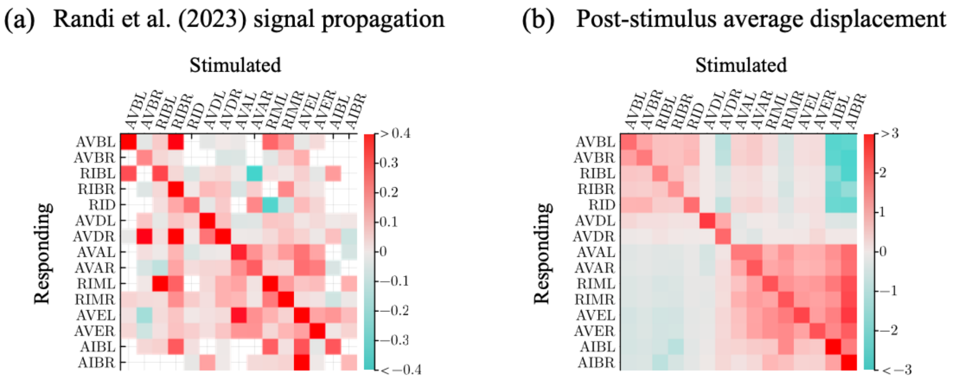

In the added section "Ablation of core neurons", we analyze the contribution of each core neuron by simulating the model with signal input this time and ablations to the core set. In Figure 6(a-c) we show the simulated core neurons when the RIB and AIB neurons are ablated, which we found to be the most crucial neurons for maintaining the original dynamics.

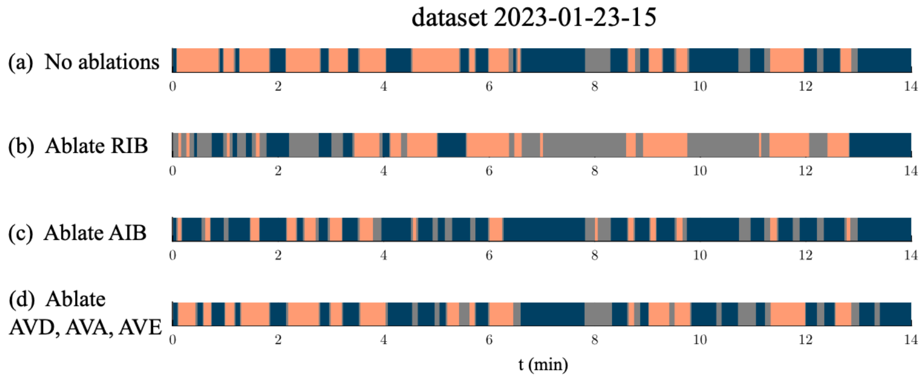

3. The time series used in Figure 5 is the actual data taken from Atanas et al., and it is unclear how the ODE model proposed by the authors contributes to this analysis. In addition, it is known that the duration of forward movement roughly determines whether the animal is dwelling or roaming, so it is necessary to show whether the proposed model can reproduce the distribution of the duration of forward movement.

Response:
As the reviewer pointed out, the original Figure 5 did not contain anything from the simulations and the model did not contribute anything new beyond what was previously known.
We have changed the section, "Behavior over long durations: roaming vs dwelling", and the accompanying figure, Figure 9. Figure 9 (the new Figure 5) now contains behavior timeseries from the simulations rather than the original data. The random paths are reconstructed from the simulations now (rather than the original data), and show that the simulations produced by the

model produced different distributions that resulted in changes to the amount of space explored in the random path simulations. Figure 9(b) now shows the simulation and random path for dataset 2023-01-23-15.

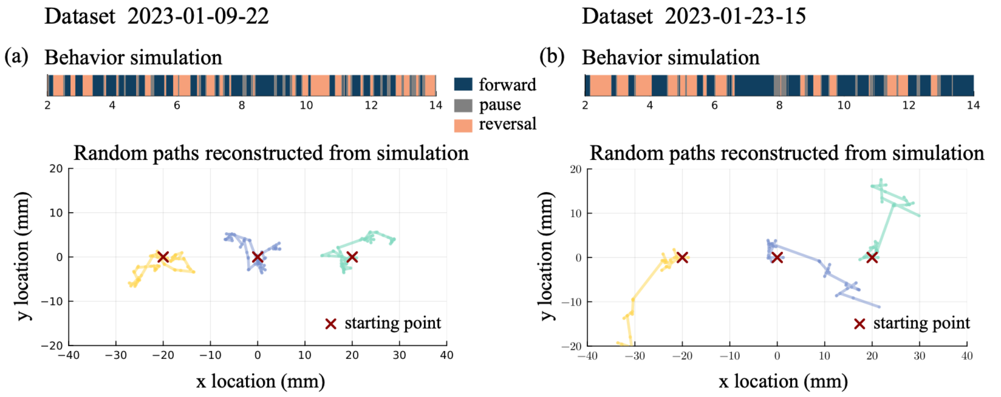

Fig 9. (a) Behavioral state timeseries from the model simulation using dataset 2023-01-09-22 [1], and three locomotion paths simulated from this behavioral timeseries. The simulation procedure is described in the Methods. (b) Analogous results using dataset 2023-01-23-15 as input for the simulation.

4. Regarding to Figure 6: The weight from ASEL/R to the downstream circuit is just not visible. In the experimental dataset, there is no obvious input to ASEs, so it would be difficult to correctly estimate the weight to AIB. The apparent weight from ASER to AIB is already report (Sato H., et al., 2021, 10.1016/j.celrep.2021.109177).
Overall, it would be difficult to analyze responses to stimuli based on Atanas' dataset. It seems that a detailed analysis of the heat avoidance experiment conducted by Atanas et al. would be possible, but it is not mentioned at all in this paper. AWC is activated very transiently by thermal stimulation (Figure 7 in Atanas 2023). However, since other thermosensitive neurons are also activated (or inhibited) at the same time by thermal stimulation, it seems difficult to correctly estimate the effect of AWC alone.

Response:
We eliminated the original Figure 6 and accompanying section and replaced this analysis with a more systematic and widespread perturbation and ablation analysis.

The section “Promoter and suppressor signal neurons” (pg. 11) with Figure 5 shows the impact on the switching dynamics of setting to zero the most relevant signal neurons.
The section “Replicating the impact of heat shocks” (pg. 14) with Figure 8 shows the impact of perturbing heat sensitive signal neurons.

5. Regarding to Figure 7: If you want to predict synaptic polarity, you need to refer to the actual measurement values (Randi F., et al., 2023, Nature). The references listed are only guesses and

are weak as evidence. The fact that there seems to be no relationship between the number of synapses and the weight of synaptic connections is already mentioned in several papers including the NeuroPAL paper (Yemini E., et al., 2021, Cell). Also, since there is a strong correlation between neural activity, it seems necessary to consider multicollinearity for parameter estimation. If you simply estimated the parameters from a small number of real data without taking some measures, such as dimensionality reduction, the estimated values will be inaccurate. You might also want to check the sloppiness (identifiability) of the parameters.

Response:
We added section "Signal propagation maps" and Figure 7 in which we simulate the Randi et al. (2023) signal propagation experiment using our model. We compared our resulting signal propagation matrix to the experimental data from Randi et al. (2023) and offered an explanation for the discrepancy between the two. Because the relationship between functional connectivity (signal propagation) and anatomical synaptic weights is not straightforward we did not compare the functional weights to the anatomical synaptic weights in Figure 10.

In the text we added to the section "Challenges in building a mechanistic network that is data-driven, biophysical, and analyzable" the following (pg. 18):
"Additionally, there is not a clear relationship between functional connectivity and anatomical connections \cite{yemini_neuropal_2021}. Because we cannot infer synaptic signs and weights from signal propagation data, as is available from Ref.~\cite{randi_neural_2023}, we do not include the Figure~\ref{fig:Randi_weights} functional weights in the comparison of polarities."

To address the possibility of achieving more reliable and higher accuracy models we added the following text to the Section "Ongoing and future work" (pg. 18):

"On the technical side:

Although data availability is a limitation, using advanced statistical methods with the existing data may result in more accurate and reliable model fits. The identifiably analysis literature includes methods for fitting model parameters while also proving that the model fit is unique and accurate \cite{chis_structural_2011, balsa-canto_iterative_2010, grewal_identifiability_1976, saccomani_daisy_2008, ovchinnikov_input-output_2023}.
Identifiability has typically been achievable for smaller systems with well-defined terms and highly accessible measurements; it has not been generally established for larger nonlinear systems with few restrictions on terms. A useful future direction would be to explore the extent to which identifiability is achievable for neuronal systems given that the graph structure and known neuronal mechanisms will significantly constrain model possibilities."

Minor

6. Text in the legend of figure 1 contains more than legend.

Response:
We removed the last two sentences of the figure caption as these sentences were not describing the elements in the figure.

7. In figure 3b, the authors estimate the behavioral labels based on the difference between the average neural activity F(t) and R(t). This method is need to be validated. Since the behavioral labels of the real data can be estimated using the same method, it is necessary to show whether they match the real data (velocity in the dataset).

Response:
To the Appendix, we added a section titled "Inferring behavior from core neuron activity" (pg. 27) with Figure 16 that describes how we computed a proxy for behavior from the real and simulated core neuron data. We compared our proxy value for behavior to the true behavior, computed from the true velocity measure.

8. In figure 3d, the distribution of RID appears to be different between the real data and the model. Since the distribution across all time is affected by the ratio of different behaviors in each trial, it may be necessary to show a stratified histogram for each behavior label and discuss the consistency.

Response:
Dividing the sequences up by behavior would result in all the forward/reversal neurons being either on or off during most of the period with some variability. The pdf of each neuron deviates from the true distribution and this difference can be measured by using the KL divergence metric. Given the simplifying assumptions made in our model and the fact that the distributions were not used to fit the model, we expect there to be some moderate differences between the distributions. In future work, the KL divergence between distributions could be used as an additional metric in the loss function to fit the model.
We comment on the distribution differences and the potential use of the KL divergence metric in the section "Higher order statistics" on page 9.

9. Regarding Figure 5: dwelling and roaming are things that switch over time within the same individual, and they change in about 10 minutes (Flavell S.W., et al., 2013, Cell, doi: 10.1016/j.cell.2013.08.001). The data is obtained from Atanas 2023, but are these really dwelling and roaming? It is possible that you are simply looking at individual differences.

Response:

The datasets that we use as examples of dwelling and roaming in section "Behavior over long durations: roaming vs dwelling" and Figure 9 (previously Figure 5) are not experimentally labeled as dwelling and roaming and there is no definitive experimental evidence that they fall into one of these two categories. The differences observed could be individual differences between animals and do not, in fact, fit the criteria to be labeled as "dwelling" or "roaming". We have modified to text to clarify that the two behavioral sequences shown in (a) and (b) are not previously defined examples dwelling and roaming but rather produce paths the explore smaller or larger regions surrounding the starting point. The paths that stay closer to the starting point may fall into the category of dwelling while the paths that move far from the starting point may fall into the category of roaming. (Text changes are shown in blue on pgs. 14-16).

10. Figure 11 may be more appropriate as a supplementary figure.

Response:
We moved Figure 14 (previously Figure 11) to the Appendix. We added a section to the Appendix describing Figure 14 called "Additional simulations".

11. Since there are comprehensive neural and behavioral models such as MetaWorm (currently BAAIworm, https://doi.org/10.1038/s43588-024-00738-w), comparisons with these models may be informative.

Response:
As pointed out by the reviewer, we missed an interesting and relevant new model --- the BAAIworm model by Zhao et al. (2024). We have changed the text in "Relation to existing literature" (pg. 17) to summarize the part of this model that is related to the model presented in this manuscript.

"A publication that appeared recently reproduces realistic \textit{C. elegans} forward motion towards an attractor with a biomechanical body-environment model \cite{zhao_integrative_2024}. BAAIWorm is an integrative model that uses a neural network to control muscles that actuate the body-environment component of the model \cite{zhao_integrative_2024}. The neural network component is biophysically detailed with many parameters fit using machine learning and with an emphasis on its ability to produce activity that results in coordinated forward movement via the muscles and body."

We have also added a comparison of our work:
In contrast to Ref.~\cite{zhao_integrative_2024}, the model presented here posits an explanation for how the premotor network integrates sensory information to produce realistic switching dynamics --- as observed in whole-brain imaging data --- which results in forward/reversal

locomotion sequences. We formulated a parsimonious model with fewer parameters that could be fit to datasets of neural activity and analyzed in a transparent manner.

12. The conversion between membrane potential and GCaMP values seems to be based on the assumption that the binary state transitions seen in GCaMP measurements are similar to the stationary values of the I-V curve. If there are any evidence that justify this assumption, it would be better to cite the literature.

Response:
We have modified the text in the Methods "Intrinsic dynamics" section to further justify our choice of functional form for the intrinsic dynamics on pages 20-21 (text change shown in blue).

Reviewer 3

1. The main limitation I see in this approach is that it is not clear what, if anything, specific to the core circuit actually accounts for the ability to produce similar dynamic behaviour. That is, it seems possible that a completely generic neural network could be tuned/trained to produce the appropriate (simple) output (a dynamically switching tendency towards forward or backward motion) given the very rich input information.

Response:
While many different types of networks could produce the stochastic switching sequences that are the output of this model, our goal in creating this model was to more specifically determine how the core circuit in *C. elegans* produces the switching sequences, to study how biology is connected to function for this particular organism.

For example, in the new section "Promoter and suppressor signal neurons" we identify the neurons whose activity is potentially the most relevant for initiating different types of switches.

In the new section "Ablation of core neurons", we identified, out of all the reversal neurons, reversal neurons AIB are the most crucial for maintaining the switching dynamics and this aligns with previous findings. If the model aligns closely enough to the true structure, then it can be used to explain the mechanism behind experimental results.

2. The presented results make a small start towards exploring this by examining the circuit performance with either gap junctions or synaptic connections left out, and this is the most interesting part of the paper. But a much more thorough examination is needed, e.g., to see what is the minimal circuit that can produce realistic results, to ablate individual neurons in the circuit (not just the inputs) and test the consequences, and to establish what happens as the parameter values are varied.

Response:
We have made our analysis and examination of the model more extensive with the additional sections "Promoter and suppressor signal neurons", "Ablation of core neurons", "Signal propagation maps", and "Replicating the impact of heat shocks".
The additional results are shown in Figures 5, 6, 7, and 8.

In these sections we show what happens when we both eliminate and perturb core and signal neurons. We were able to identify the most vital neurons for different behaviors, such as producing a switch or integrating information from incoming signals. There are potentially many different circuit structures that could produce the output of interest, however, for this study we limited our model and analysis to the structural and biological constraints that we found in the experimental literature.

3. The model also seems to make a number of other serious compromises with respect to realism, such as no recurrency from core to signal neurons, eliminating some potentially relevant neurons due to lack of data, not modelling motor output or motor feedback, ignoring neuromodulation, etc. Of course, any practical model has to make compromises to be tractable, and some of these limitations are discussed appropriately, but they also limit what can actually be learnt from the model. For example, while it is interesting to see the predicted polarity for neural connections obtained from the data fitting approach, and to discuss how these align with or contradict previous accounts, it seems likely the fitted connection weights, and their polarity, could be very different if any of the above factors were included.

Response:
The reviewer is right in pointing out that many compromises are made in the model. Some compromises are made to achieve simplicity while other compromises are due to a lack of information or data. We view this study as a first step in building a dynamical model that strikes a compromise between analyzability and realism. Including more factors and parameters could certainly change the connection weights and their polarities.
We have added a description of how the model could be improved in section "Ongoing and future work", subsection "On the technical side" (p. 20).

4. I also felt the paper overall did not give a sufficiently deep embedding of how this work relates to the extensive range of previous models of this system. Instead these tended to be described somewhat briefly and dismissively.

   Response:
   We have performed literature searches and believe we have included a representative sample of existing models. Most of the models are phenomenological in nature; we appreciate that such models are important, but they serve very different purposes than ours. Phenomenological studies aside, there were not many papers that focused on dynamics. There was, however, one paper, brought to our attention by Reviewer 1, that we had omitted: the reference on the BAAIworm model by Zhao et al. (2024). We have added that reference, and have modified the Discussion section "Relation to existing literature" (pgs. 17) with text changes shown in blue to include a discussion of that paper. We also add a more extensive description of how the purpose and capabilities of our model differ from previous models (pg. 17).

5. Abstract:

   "minimally parameterized, biophysical dynamical systems mode" - I am not sure that I agree that the degree of tuning to data actually used to set the parameters here should be described as "minimally parameterized". It is also not fully evident in what sense the model is "biophysical". The only support is in the claim (in the methods) that the cubic approximation for the intrinsic dynamics of the core neurons is similar to the form of models (e.g. Fitzhugh-Nagumo and Hindmarsh-Rose) of neural voltage dynamics. I agree the approximation does capture appear to capture the key properties, but this is not the same as having a biophysical justification for the elements of the equation.

Response:
We agree that it is more accurate to describe our model as "biology-based" rather than biophysical (as not all components are biophysical). We have made the changes accordingly in the abstract and throughout the paper.
We have also included a more extensive justification for the form we posit for the intrinsic dynamics in the Methods "Intrinsic dynamics" section on pages 20-21.

6. Introduction:

   It seems unnecessary to characterize the current state of understanding so negatively, e.g. "The neural mechanisms responsible for producing different behaviors and inducing transitions are not well understood, even in the simplest of organisms". "relatively little is known" "the mechanisms that govern their collective activity remain to be understood". I would prefer the authors to provide a clear outline of what is known, what has been hypothesized, and how the problem has been approached to date. From the introduction, a

naive reader would be unaware of the extensive range of previous models of this system, as these mostly are referenced only in the discussion.

Response:
We appreciate your concerns and have modified the language in the Introduction (in blue). We agree also that it is important to provide an outline of what is known and what is not, and this is what we have tried to do in the second and third paragraphs of the Introduction. For example, we wrote:

"To some extent, experimentalists have demystified how sensory neurons respond to stimuli and how motor neurons coordinate muscle activity \cite{goodman_how_2019, bargmann_chemosensation_2006, zhen_c_2015, wen_proprioceptive_2012, gao_excitatory_2018}, but relatively little is known about how the intermediary interneurons operate collectively to make behavioral decisions."

"Individual neurons that promote forward and reversal movements have been identified, but the mechanisms that govern their collective activity remain to be understood."

"The connectome \cite{white_structure_1986} provides detailed, quantitative information on connections between pairs of neurons in the entire organism, but anatomy alone -- without physiology or dynamics -- does not predict activity."

"Whole-brain imaging data \cite{kato_global_2015, atanas_brain-wide_2023} connects neuronal activity to behavior, yet does not explain how activity patterns are generated."

7. Line 26 on, it is good in principle to set out your modelling criteria, but other criteria might be advanced, so why are these chosen?

Response:
We did not mean to suggest those are the only valid criteria, and have changed the phrasing there to state that (i)-(v) are *our* guiding principles.

8. Line 107 on, the explanation for selecting neurons seems somewhat vague. Was there an explicit criteria, e.g., neurons shown to produce locomotion when individually activated ("command neurons")? Neurons one synapse away from motor neurons ("premotor neurons")?

Response:

Our choice of core neurons is somewhat arbitrary, guided by what is known from experiments, connectivity pre and postsynaptically with the core group, and practical considerations such as data availability as we have discussed. We know of no clear experimental information on the set of neurons that produce locomotion when individually activated, and the set of neurons one synapse away from motor neurons is very large. The premotor neurons are highly interconnected with the remainder of the network and do not form a clearly distinct community in the network from a graph theory perspective.

9. Equation 1, the summation terms seem to imply that the inputs are the (W_ij) weighted gap junction inputs from both core and signal neurons, and the (A_ij) weighted inputs from both core and signal neurons; this is also what Fig 2a and 2b seem to show. Yet the text on lines 147-150 describes these terms in the equation as the “influence from all core neurons”. This creates confusion as I read the following parts under the assumption that A_ij is fitted to the data for the core neurons only, but (I believe) in fact it is also fitted to the signal data. It seems this could be sufficient to explain the matches in switching dynamics and higher order statistics shown in the results, irrespective of the structure of the core circuit.

Response:
The A_ij and W_ij are from all core and signal neurons. That is now fixed.

10. The differential effects of synapses vs. gap junctions seems potentially interesting. But could the correct dynamics be recovered if the parameters were refitted for the circuit without synapses/gap junctions?

Response:
Both synapses and gap junctions are extremely substantial parts of the *C elegans* connectome. Deviating from the connectome in such a major way from the start while fitting the network with *C elegans* behavioral data is tantamount to requiring a different organism to exhibit the same behavior as the *C elegans*. All parameters will be affected, the fit will likely be poor, and the resulting network hard to interpret. By staying as faithful as possible to the biology and changing e.g. one neuron (or small group of neurons) at a time as is done in ablation experiments, we hope to investigate the influence of that specific neuron or group of neurons.

11. Behaviour over longer time durations: I found this hard to follow, particularly how it relates to the model provided here. It seems hardly necessary to provide a model to support the inference that more frequent switching results in shorter runs, and hence less dispersal. This part of the work seems unnecessary to include in the paper.

Response:
This point was not made correctly in the original submission. We have now fixed it to show how model outputs can simulate roaming and dwelling behaviors. We would like to include this result

as it treats an aspect of behavior not touched upon earlier. This section of the paper highlights that are model produces longer-term behaviors such as roaming or dwelling. These longer-term behaviors are not reproduced by models whose behavior output is limited to forward locomotion.

12. Lines 359-361, rather than dismissing these mathematical models, it would be more interesting to discuss if the current model gives more support to one of these abstractions over another.

Response:
It was certainly not our intention to dismiss the mathematical (phenomenological) models; they provide analogies and help connect phenomena with math concepts. These models are descriptive, and are often built to illustrate a specific phenomenon. There is not enough similarity in structure or output between our model and these models to provide conclusive support for one over another. Many of these models also differ in terms of their objective and are not necessarily in conflict with one another.

13. Line 411, can you comment more on how the accuracy of the model could improve. E.g. In what way would you test for a better fit to the data than that shown in fig 3 for the current model?

Response:
In Discussion, under "Challenges in building a mechanistic network…", we have expanded the paragraph on model limitations and added comments on how the model could be improved. Additionally, a specific proposal for model improvement is added in "Ongoing and Future work", under "On the technical side".
