# Supplementary material for: A data-driven biology-based network model reproduces C. elegans premotor neural dynamics
Source: PLoS Comput Biol. 2025 Dec 29;21(12):e1013818. doi: 10.1371/journal.pcbi.1013818 (PMC12768384; doi:10.1371/journal.pcbi.1013818)
Supplement: Attachment — Submitted filename: Response to Reviewers2.pdf [file pcbi.1013818.s002.pdf]

Dear Dr. Barbara Webb and Dr. Hugues Berry,
PLOS Computational Biology

RE: Manuscript # PCOMPBIOL-D-25-00009, "A data-driven biophysical network model reproduces *C. elegans* premotor neural dynamics"

We thank the reviewers for their follow-up evaluation of our manuscript. The reviewers highlighted additional issues that we needed to address. We believe that the changes we have made in response to the reviewers' comments have further improved the manuscript and we hope that it will be accepted for publication in PLOS Computational Biology.

Sincerely,

Megan Morrison and Lai-Sang Young

---

**Reviewer #1:**

*The authors conducted additional analyses and clarified key features of the model. I am generally satisfied with the manuscript revisions addressing my comments, but I would like to add several minor comments.*

1. *Regarding the section "Promoter and suppressor signal neurons" added by the authors, the forward-reverse switching in their model appears to be highly dependent on input signals. The authors' response includes the following description:*

*" In the absence of any stimulus the neurons stabilize at different values with the forward neurons more active than the reversal neurons. Perturbing each neuron induces changes to the state of downstream neurons, as shown in Figure 7(d), emulating the experimental results reproduced in Figure 7(c). "*

*Including such descriptions not only in the response but also within the main text would likely facilitate readers' understanding of this model. It would also be beneficial to discuss whether experimental evidence supports these findings.*

Response:

On page 14 we include the description:

"In the absence of any stimulus, the neurons converged to a fixed point of the undriven core neuron dynamics. The forward neurons stabilized at higher values than the reversal neurons, indicating that the

core network contains bias in favor of forward locomotion.  Ref.~\cite{kawano_imbalancing_2011} also found that the \textit{C. elegans} neuronal network is biased in favor of forward-circuit activity."

*Furthermore, if possible, I would like to see a description of what insights this model provides regarding the switching mechanism in real worms. Also, Toyoshima 2024 (doi: 10.1371/journal.pcbi.1011848) constructed a model focusing on neural dynamics based on whole-brain imaging data and structural connectivity, similar to this paper. However, in that model, switching of command interneuron activity occurs solely due to neural activity noise, without requiring any specific stimulus input. If possible, it might be worthwhile to discuss why this difference arises.*

Response:

On page 20, under summary and conclusion we add the following observation about switching mechanisms:

"Model results suggest that, in the real worm, much of the seemingly random switching is in response to an array of inputs distributed across a large number of sensory and interneurons, some more influential than others.  We hypothesize also that reverse motion is not antithesis to forward motion, rather it may serve as a brake to forward motion."

On pages 18 we added a reference to Toyoshima 2024 and compared it to our model:

"Ref.~\cite{toyoshima_ensemble_2024} constructed a model from whole-brain imaging data that was collected while the worm was subjected to sensory stimuli in the form of periodic changes in NaCl concentration, which elicited a response in some sensory neurons.  While locomotion neurons receive input from all of their presynaptic neighbors, Ref.~\cite{toyoshima_ensemble_2024} found that the neural activity of the premotor neurons resided in separate motifs from the motifs capturing the sensory response to NaCl concentration changes. From this, they deduced that noise was necessary to produce realistic premotor activity.  Our model leads to a different hypothesis: stochastic switching in our model occurs as a direct consequence of input from a (large) group of sensory and interneurons we call ``signal neurons".  Our findings are not necessarily in conflict, as the signal neurons that act as stimuli in our model are a more comprehensive set of stimuli than the neurons that responded to NaCl concentrations in Ref.~\cite{toyoshima_ensemble_2024}."

2. *The response to the issue raised by Reviewer 3 in comment 4, in which the introduction does not address the fundamental differences from past similar mathematical model papers, appears insufficient. The revisions now include two sentences: "There is also a substantial theoretical literature, much of it consisting of phenomenological studies (see Discussion). With all of these available tools, we believe it is*

*time to attempt a more systematic understanding of the neural mechanisms that drive function." However, the term ”phenomenological" alone does not clarify the specific strength of this paper compared to these previous studies, making the explanation insufficient. These differences should be properly explained in the introduction, not merely touched upon in the discussion.*

Response:

We have added a section to the Introduction titled **Previous Models (pages 3-4)** in which we describe the features of previous models.

We clarify what we mean by phenomenological:

“Many existing models fall into one of the following broad categories: \textit{Phenomenological models} aim to describe or clarify empirical observations by connecting them to mathematical frameworks without claiming to reproduce biological mechanisms. \textit{Mechanistic} and \textit{data-driven models} make more direct connections to the anatomy and physiological data of the organism.”

We note the following major drawback of phenomenological models:

“These models connect switching phenomena to mathematical frameworks, but it is hard to draw biological inferences from them as they are too far removed from neuroanatomy.”

We added additional references:

“Other recent work includes data-driven models introduced in Refs.~\cite{toyoshima_ensemble_2024} and~\cite{creamer_bridging_2025}. Ref.~\cite{toyoshima_ensemble_2024} applied independent component analysis with time-delay embedding to whole-brain imaging data to decompose the activity into meaningful components. Ref.~\cite{creamer_bridging_2025} assessed the extent to which the connectome captures signaling in the network and found that physical connections capture causal relationships.”

Under Discussion – Relation to existing literature (page 17) we note a major difference between our model and previous mechanistic models.

“Our data-driven, biology-based network model presents a dynamical picture different from those in Refs.~\cite{zhao_integrative_2024}, \cite{kim_neural_2019}, and \cite{gleeson_c302_2018}, where biophysical models of neurons respond to stimulus but do not produce activity that is qualitatively similar to whole-brain imaging activity and furthermore do not reproduce the stochastic switching phenomena.”

3. *The followings seem typos.*

*Around Line 406, "Signal input from dataset 2023-01-23-15 produces high frequency switches, resulting in shorter forward runs and reversals (Fig. 9(a))." The dataset number appears to be incorrect.*

*Around Line 569 "identifiably" -> "identifiability"*

Response:

dataset 2023-01-09-22, incorrect dataset reference fixed.

Identifiability typo fixed.

4. *The code is in its old state*

Response:

We have updated the GitHub repo to include the new code that generates the new figures. The figures associated with each notebook are now listed in the README file.
